# Trabeculated (non-compacted) and compact myocardium in adults: the multi-ethnic study of atherosclerosis

**DOI:** 10.1186/1532-429X-14-S1-O86

**Published:** 2012-02-01

**Authors:** Nadine Kawel, Marcelo Nacif, Andrew E Arai, Antoinette S Gomes, William Hundley, Craig Johnson, Martin R Prince, Brandon Stacey, Joao A Lima, David A Bluemke

**Affiliations:** 1 Radiology and Imaging Sciences, National Institutes of Health, Bethesda, MD, USA; 2 National Institute of Biomedical Imaging and Bioengineering, National Institutes of Health, Bethesda, MD, USA; 3 National Heart, Lung and Blood Institute, National Institutes of Health, Bethesda, MD, USA; 4 Department of Radiology, UCLA School for Medicine, Los Angeles, CA, USA; 5 Department of Internal Medicine / Cardiology, Wake Forest University, Winston-Salem, NC, USA; 6 Collaborative Health Studies Coordinating Center, University of Washington, Seattle, WA, USA; 7 Cornell and Columbia Universities, New York, NY, USA; 8 Division of Cardiology, Johns Hopkins University, Baltimore, MD, USA

## Background

A high degree of non-compacted (trabeculated) myocardium in relationship to compact myocardium (T/M ratio >2.3) has been associated with a diagnosis of left ventricular non-compaction (LVNC). The Multi-Ethnic Study of Atherosclerosis (MESA) is a population-based longitudinal study initiated in July 2000; with 6814 participants (45-84 years, 3601 women) free of recognized cardiovascular disease at enrollment. The purpose of this study was to determine the normal range of the T/M ratio in MESA and to examine the relationship to demographic and clinical parameters.

## Methods

The thickness of trabeculation and the compact myocardium were measured in eight regions of the left ventricle on long axis cardiac magnetic resonance (CMR) steady-state free precession cine images in 1000 randomly chosen participants of the “MESA 5” follow-up cohort (551 women; 68.1±8.9 years) and T/M ratios were calculated.

## Results

**Figure 1:**
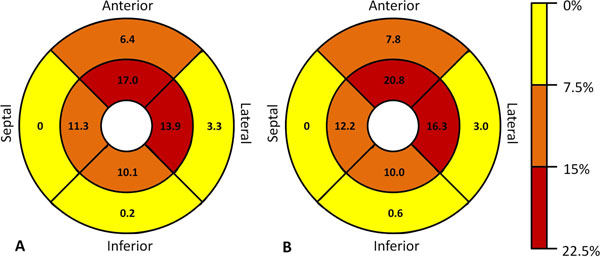
Percent subjects with a T/M ratio >2.3 per region at the mid-cavity level (outer circle) and the apical level (inner circle) of the entire cohort (A) and the subset of subjects free of cardiac disease (B).

**Figure 2:**
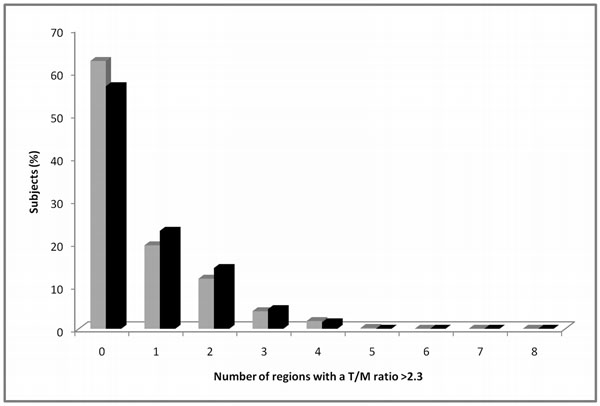
Percent of subjects of the entire cohort (grey bars) and the subset of subjects free of cardiac disease (black bars) with a T/M ratio >2.3 in 0 to 8 regions per subject. T/M ratio = thickness of trabeculation / thickness of compact myocardium.

In a subset of 323 participants free of cardiac disease and without known LVNC, 140 (43%) had a T/M ratio >2.3 in at least one region (Figure 1) while 20/323 (6.2%) participants had a T/M ratio >2.3 in more than two regions (Figure 2). 62/323 (19%) had a T/M ratio >2.9 in one region. Multivariate linear regression model revealed no association of age, height and weight with the maximum T/M ratio and trabecluation thickness in participants free of cardiac disease (p>0.05). Maximum trabeculation thickness was associated with Chinese and African American ethnicities and male gender (p<0.05; β=1.5mm, 1.3mm and 1.1mm, respectively). In participants free of cardiac disease, maximum trabeculation thickness and T/M ratio were associated with LV end-diastolic volume (p<0.0001; β=0.03mm/ml and β=0.01/ml, respectively) and end-systolic volume (p<0.001; β=0.06mm/ml and β=0.03/ml, respectively) in adjusted models. Further, there was a negative association of LV ejection fraction with maximum T/M ratio (p=0.044; β=-0.02/%). There was no association of maximum T/M ratio with hypertension or myocardial infarction (p>0.05) in adjusted models of the entire cohort (n=1000). Values for T/M ratio depend on measurement technique: At the apical level median T/M ratios derived from measurements of short axis images were significantly less than the values obtained on long axis images (p=0.017).

## Conclusions

Results of the current study suggest a reevaluation of the current CMR criteria for LVNC using a higher cut-off for T/M ratio and including the number of affected regions. A uniform definition of measurement technique is necessary.

## Funding

This research was supported by contracts N01-HC-95159 through N01-HC-95169 from the National Heart, Lung, and Blood Institute.

